# Different Genotype Distribution of Human Papillomavirus between Cervical and Esophageal Cancers: A Study in Both High-Incidence Areas, Xinjiang, China

**DOI:** 10.1155/2020/7926754

**Published:** 2020-12-02

**Authors:** Yi Zheng, Yage Fan, Yan Zeng, Shiyu Liu, Limin Gao

**Affiliations:** ^1^Department of Gastroenterology, First Affiliated Hospital, School of Medicine, Shihezi University, Xinjiang, China; ^2^Shihezi University School of Medicine, Xinjiang, China; ^3^Key Laboratory of Xinjiang Endemic and Ethnic Disease & Department of Biochemistry, School of Medicine, Shihezi University, Xinjiang, China; ^4^Department of Gynecology and Obstetrics, First Affiliated Hospital, School of Medicine, Shihezi University, Xinjiang, China

## Abstract

The aim of this study is to reveal the certain human papillomavirus (HPV) genotype distribution between cervical cancer and esophageal cancer in the both high-incidence geographic regions. For this study, we collected and detected the infection of HPV in 120 paraffin-embedded esophageal tissues and 152 paraffin-embedded cervical tissues, respectively. The esophageal tissues include 40 normal epithelium (ENOR), 26 dysplasia (DYS), and 54 invasive squamous cell carcinoma (ESCC). The cervical tissues consisted of 40 normal epithelium (CNOR), 53 intraepithelial neoplasia (CIN), and 59 invasive squamous cell carcinoma (CSCC). Both esophageal and cervical tissues collected in this study came from the same area, in which both the ESCC and CSCC were in high incidence, Xinjiang province, China. HPV GenoArray test kits were served to analyze the HPV infection. The result shows that among the 59 CSCC tissues, the total infection rate of HPV was 98.3% (58/59). The positive rate of HPV-16 infection was 63.8% (37/58). It indicated that HPV-16 is the most common infection among all of the high-risk HPV. The multiple infection rate was 19.0% (11/58). Among the 54 ESCC, a total of 7 genotypes were detected. The total infection rate of HPV was 61.1% (33/54). The positive rate of HPV-16 infection was 63.6% (21/33). The multiple infection rate was 6.1% (2/33). Our result shows that high-risk-type HPV-16 was associated with both cervical cancer and esophageal cancer, which play a role in the high-incidence area in Xinjiang. We hope that our results could point out the direction for the treatment strategy of HPV-associated cancer, cervical cancer, and esophageal cancer and for the application of HPV vaccines in the future.

## 1. Introduction

Human papillomavirus (HPV) is an extremely common pathogen for human infection and can cause many benign and malignant diseases, including cervical, anal, vulvar, penile, and oropharyngeal cancers [1]. Meanwhile, its genotyping is closely related to pathogenicity. There are more than 100 subtypes of HPV, which are divided into high risk and low risk based on their propensity to cause cancer. It was reported that high-risk human papillomaviruses (HR-HPVs) brought about more than 600,000 cancers in 2008, which bring huge pressure and enormous challenges for public health [2]. Infection by high-risk anogenital HPV is a necessary cause of cervical cancer (CC) [3]. HR-HPV-associated cancers closely correlated with different anatomic sites, geographical areas, and smoking status, leading to almost 100% cervical cancer, 88% anal cancer, and less than 50% lower genital tract and oropharyngeal cancer [4].

However, due to healthcare disparities, unbalanced economic development, and unhealthy lifestyle, the developing countries have a higher incidence and mortality rates of cervical cancer. According to global cancer statistics in 2018, the number of new cases and deaths of the cancer reached 315,346 and 168,411, respectively, in Asia. In addition, the incidence and mortality rates were 3 to 5 times higher than Northern American and Australia/New Zealand [5]. In 2018, it was evaluated that there were 106,430 new cases and 47,739 deaths all over the country [6]; cervical cancer is regarded as one of the top six cancers among women in China, especially in parts of rural area [7]. For example, in the southern part of Xinjiang, the morbidity (up to 590/100,000) and mortality rate of cervical cancer is the highest in the whole country [8]. It is worth nothing that the incident and death rates of esophageal cancer are also high in these regions.

Esophageal cancer is the ninth most common malignancy around the world; multiple factors including drinking, smoking, eating habits, poverty, and history of head and neck cancer are involved in its complex and multistage mechanism [9]. What is more, studies in the past 20 years have shown that the HPV plays a crucial role in the development and progression of esophageal cancer as a cofactor or oncogene. But its idiographic action mechanism is to be addressed. In China, infectious agents contributed more than one-quarter of the overall cancer cases [10]. The infection agents have been recognized as either direct carcinogens or promoters in esophageal carcinogenesis. HPV has been suggested as distinct possible cause of esophageal carcinogenesis [11].

The study of HPV genotyping in invasive cervical cancer shows that the distribution and prevalence of HPV vary by geographic region and race [12]. Located in the northwest of China, Xinjiang has long been inhabiting 47 ethnic groups such as Han, Uygur, and Kazak. Because of a large population and complex landforms, both cervical cancer and esophageal cancer were in high incidence in this area. It has been reported that the incidence of cervical cancer ranges from 459/100,000 to 572/100,000, which is significantly higher than the national incidence (14.6/100,000) [13]. Esophageal cancer also has a high incidence in Xinjiang, up to 150/100,000. However, the certain difference of HPV genotype distribution between these two cancers in the same geographic regions is not clear. In this study, we used PCR and Gene Chip to detect and genotype the HPV, respectively, among paraffin-embedded esophageal and cervical tissues to find the difference of HPV infection between cervical cancer and esophageal cancer, hoping that our results could point out the direction for targeted therapy of HPV-associated cancer, cervical cancer, and esophageal cancer and for the application of HPV vaccines.

## 2. Materials and Methods

### 2.1. Study Subjects

#### 2.1.1. Source of Cervical Samples

All of the paraffin-embedded cervical tissues were from the First Affiliated Hospital of Shihezi University, Xinjiang, China; healthy control and patients with cervical precancerous lesions were selected randomly from who underwent colposcopy examination, and patients with CSCC received surgical treatment. All cases were confirmed by histological examination of tissue from biopsy or resected specimens during 2010-2018. The study group included a total of 152 subjects and being composed of a control group of 40 normal cervical epithelium (CNOR), two study groups of 53 cases and 59 cases diagnosed with intraepithelial neoplasia (CIN), and 59 invasive squamous cell carcinoma (CSCC), respectively.

#### 2.1.2. Source of Esophageal Cancer Tissues

All of the tissues were paraffin-embedded esophageal tissues which were collected from the First Affiliated Hospital of Shihezi University, Xinjiang; Friendship Hospital of Yili, Xinjiang; Xinjiang Production and Construction Corps Agriculture 4th Division Hospital; and People's Hospital of Yili, Xinjiang, during 2010-2018. A total of 120 cases of esophageal tissues are from the Kazak people of Xinjiang. All of the cases were observed under an optical microscope, including 40 normal epithelium (ENOR), 26 dysplasia (DYS), and 54 invasive squamous cell carcinoma (ESCC). Healthy subjects and patients with esophageal precancerous lesions were confirmed by early esophageal cancer screening. Esophageal biopsy tissues were divided into normal and DYS depending on cellular morphological changes and tissue architecture. The ESCC tissues came from the patients who underwent the surgical treatment.

### 2.2. Methods

#### 2.2.1. Sample Collection

Biopsy materials and surgical specimens were collected and processed by standardized methods to minimize the possibility of HPV contamination in the environment. Both the esophageal and cervical tissues were fixed with formalin and embedded in paraffin.

#### 2.2.2. Histopathological Examination

The cervical intraepithelial neoplasia terminology was used for classification of cervix biopsy tissues. The histopathological examinations and TNM staging of cervical cancer specimens and esophageal cancer specimens were performed according to the TNM classification given by the UICC criteria of 2002 [14] and FIGO criteria of 2009 [15], respectively.

#### 2.2.3. DNA Extraction

The DNA extraction procedures in the paraffin-embedded both esophageal and cervical tissues are as follows: 10 pieces of 5 *μ*m paraffin-embedded tissues were treated with xylene for dewaxing. Then, it was added with 30 *μ*l proteinase K (20 mg/ml) and incubated overnight under 55°C. Then, DNA was extracted by the same method with cervical cells (reagents, purchased from Sangon Biotech (Shanghai) Co. Ltd).

#### 2.2.4. PCR

The relevant segment of the HPV L1 gene was amplified by general consensus primers GP5+/GP6+ (GP5+5′-TTTGT TACTG TGGTA GATAC TAC-3′, GP6+5′-GAAAA ATAAA CTGTA AATCA TATTC3′) [16]. The 268 bp fragment of the *β*-globin gene was amplified by Glob-F (5′-CAACTTCATC-CACGTTCACC-3′) and Glob-R primer (5′-GAAGAGCCAAG-GACAGGTAC-3′) to confirm the existence of human genomic DNA. Each group of PCR had negative control which was a reaction solution without template DNA. For reaction system and conditions, refer to reference [17]. Positive PCR products were used for HPV genotyping (reagents purchased from Tiangen Biotech (Beijing) Co. Ltd).

#### 2.2.5. HPV Genotyping

HPV genotypes were analyzed by commercial HPV GenoArray kit (Yaneng Bioscience (Shenzhen) Co. Ltd.) for detection and genotyping of 23 HPV types (HPV-16, HPV-18, HPV-31, HPV-33, HPV-35, HPV-39, HPV-45, HPV-51, HPV-52, HPV-56, HPV-58, HPV-59, HPV-68, HPV-73, HPV-82, HPV-6, HPV-11, HPV-42, HPV-43, HPV-82, HPV-53, HPV-66, HPV-83), including all the 13 high-risk HPV genotypes. All the operations were conducted in strict accordance with the manufacturer's agreement. The identification of the results was also based on the manufacturer's instructions. 23 HPV subtypes can be identified by HPV genotyping. All HPV subtypes were represented by Arabic numerals. The different concentrations of DNA were the reason for the different intensity of positive “blue spot.” When the DNA concentration of the sample was high, there was a “blue dot” in the internal control (IC).

### 2.3. Statistical Analyses

Data were analyzed by using SPSS software version 17.0. The Spearman correlation test was carried out to determine whether or not the HPV-positive rates were correlated with the progression of carcinogenesis. Otherwise, unconditional logistic regression was used to perform the relationship between HPV infection and disease progression; numerical data were expressed as odds ratios (OR) with 95% confidence intervals (CI). For all tests, statistical significance was defined as a two-tailed *P* value less than 0.05.

## 3. Results

Total samples were tested using the HPV GenoArray kit; 165 (60.7%) cases were positive. Among the positive samples, 37 cases of cervical and 21 cases of esophageal cancers were HPV-16 subtypes.

### 3.1. Genotypes Detected in Cervical Tissues

Of the 152 samples analyzed, 73% (111) were HPV-positive by Gene Chip. 20 of the 23 HPV genotypes, which the Gene Chip contains, were detected. The increasing risk of CIN and CSCC, respectively (CIN vs. CNOR: OR = 43.94, 95%CI = 9.44-204.56; CSCC vs. CNOR: OR = 105.56, 95%CI = 21.54-517.19) was observed. There was infection with high-risk HPV types of CIN and CSCC by 43.94-fold and 105.56-fold. There was a significant correlation between infection with high-risk HPV types and the progression of CSCC (*P* < 0.001) (Table 1).

### 3.2. Genotypes Detected in Esophageal Tissues

The detection rate of high-risk types was 76% (41/54) of 120 samples. Infection with high-risk HPV types increased the risk of DYS by 6.53-fold (DYS vs. ENOR: OR = 6.53, 95%CI = 1.57-27.21) and ESCC by 14.31-fold (ESCC vs. ENOR: OR = 14.31, 95%CI = 3.93-52.10).

### 3.3. Distribution of HPV Subtypes in 152 Cervical Samples

HPV-DNA testing including all of the 23 different HPV types was performed in a total of 152 cervical tissues. The 111 HPV-positive samples were detected (CNOR: 9, CIN: 44, CSCC: 58). The high-risk HPV genotypes account for 80.2% (89/111) of the total positive rate of cervical samples. HPV-16 was identified in a total of 64 among the 152 positive cases, which was the most frequent viral type. HPV-11 was the most frequent low-risk type; it accounts for 66.7% (10/15) of the total low-risk types (Table 2). Single-type infections were identified in 47 cases, accounting for 81%. Double infection was predominated among the multiple infections, accounting for 45.5%. The more the types of samples infected, the lower the cases detected (Table 3). Among the double infections, HPV-16 combined with other types was dominant. HPV-16 combined with HPV-33 was the most frequent form.

### 3.4. Distribution of HPV Subtypes in 120 Esophageal Tissues

Of the 120 samples analyzed, 45% (54) were HPV-positive by Gene Chip. Seven of the 23 HPV genotypes, which the Gene Chip contains, were detected. The 54 HPV-positive samples detected were as follows: ENOR: 8, DYS:13, and ESCC:33. HPV-16 was identified in a total of 30 among the 54 positive cases, which was the most frequent viral type. Four high-risk HPV genotypes were detected, with the sequence as HPV-16 (30), HPV-18 (7), HPV-59 (1), and HPV-45 (1). And the most frequent low-risk genotypes were detected (Table 2). Among the 54 positive samples, single infection accounts for 93.9% (31). Multiple infections were identified in 2 cases. All of the multiple infections are double infection.

### 3.5. Clinical Pathological Characteristics

The prevalence of high-risk HPV types in CSCC patients (84.7%) was higher than that in ESCC patients (53.7%). There was no significant difference between esophagus and cervix than in precancerous tissue.

And then, we try to analyze whether the clinical pathological characteristics were associated with both CSCC and ESCC with HPV infection. However, there was no significant difference in age between over the age of fifty (75.0) and under the age of fifty (87.2) HPV infection CSCC patients (*P* = 0.547). Similarly, there was no significant difference in ESCC (*P* = 0.625). Among the 59 CSCC samples, 13 cases were positive with HPV infection; 19 cases were positive for lymph node metastasis; the rate of HPV infection was 68.4%, but in negative lymph node metastasis, the HPV infection rate was 92.5%. However, the difference was statistically significant among lymph node metastasis (*P* = 0.044). In this study, there are no statistically significant correlations found between positive HPV infection and the age or gender of patients, tumor stage, tumor cell differentiation, or lymph node metastasis in ESCC (*P* > 0.05).

Also, there is no significant association found between infection with high-risk HPV types and gender, age, lymph node metastasis, clinical stage, or differentiation in both CSCC and ESCC patients (Table 4).

## 4. Discussion

At present, over 100 HPV genotypes have been isolated, of which 13 of them have been found to be associated with malignant tumors, including cervical cancer and esophageal cancer, and therefore were classified as high-risk types. Both of cervical cancer and esophageal cancer are in high incidence in Xinjiang. However, the certain difference of HPV genotype distribution between these two cancers in the same geographic regions is not clear. In this study, we used PCR and Gene Chip to detect and genotype the HPV, respectively, among paraffin-embedded esophageal and cervical carcinoma tissues to find the difference of HPV infection between cervical cancer and esophageal cancer, in order to provide useful reference data for the prevention and treatment of HPV-associated cancer, cervical cancer, and esophageal cancer. HPV vaccine has been applied in numerous western countries for many years, but it was approved in China in 2018. The current design and validation of vaccines are based primarily on the epidemiological background of western populations, lacking of data of the certain HPV genotype distribution in Asia; thus, the protection rate in Asian populations is relatively lower than that in western populations. This study also can provide useful data for the application of HPV vaccines in the future in Xinjiang China.

We detected 152 paraffin-embedded cervical tissues (CNOR40, CIN53, CSCC59) and 120 esophageal tissues (ENOR40, DYS26, ESCC54). A total of 272 samples were enrolled to detect and genotype HPV DNA by PCR and Gene Chip. The positive cases were diagnosed as cervical normal epithelia (*n* = 9) or cervical intraepithelial neoplasia (*n* = 44) or cervical carcinoma (*n* = 58) or esophageal normal epithelia (*n* = 8) or esophageal dysplasia (*n* = 13) or esophageal carcinoma (*n* = 33). Overall, positive cases that were infected with high-risk genotype were comparatively low in normal tissues from the cervix (5%) and esophagus (7.5%) but have a relatively higher rate in precancerous lesions and cancerous tissues, which were, respectively, CIN: 69.8%, CSCC: 84.7%, DYS: 34.6%, and ESCC: 53.7%. Our result shows that with infection of high-risk type, it was increased fortyfold and hundredfold to CIN and CSCC; it also increased thirtyfold and fiftyfold to DYS and ESCC. The infection rate of HPV and the mortality and morbidity of cervical cancer have been rising every year; age of onset has a younger trend (25- to 45-year-old) [18], which has seriously threatened women's health and lives. Early screening provides a crucial method for early detection, effective treatment, and reducing mortality. However, the rate of cervical cancer screening in China is lower than the target set by the WHO Global Monitoring Framework. According to the report [18], in 2010, only one-fifth of the women in China had been screened, and the screening rate in Xinjiang was estimated to be lower, related to the vast territory, complex population composition, language communication barriers, and fewer study sites (there are only one urban study site and five rural study sites in Xinjiang). We hope that in the future work, screening services would cover the high-risk population in Xinjiang, pay more attention on strengthening health care knowledge publicity and education, and improve the screening participation in rural areas, so as to reduce the infection rate of HPV and the incidence of cervical cancer.

Our results indicated that HPV-16, HPV-18, and HPV-56 were the most current viral type among cervical cancer in Xinjiang. A global study shows that HPV-16, HPV-31, and HPV-18 were the main types among the women with normal thinprep cytologic test (TCT) diagnosed in Europe; HPV-16, HPV-18, and HPV-33 in Asia; and HPV-16, HPV-58, and HPV-18 in South America [19]. A number of epidemiological studies have shown that high-risk HPV is the principal cause of invasive CSCC and CIN; seventy percent of these have close relationship with HPV-16 or HPV-18 [20]. Previous studies also reported the five most prevalent HR-HPV genotypes in cervical cancer in Yunnan Province, Southwest China, were HPV-16 (3.4%), HPV-56 (1.7%), HPV-58 (1.4%), HPV-33 (1.2%), and HPV-52 (0.88%) [21]. Data from ICO/IARC HPV Information Centre shows that the five most common HR-HPV genotypes in cervical cancer in China were HPV-16 (59.5%), HPV-18 (9.6%), HPV-58 (8.2), HPV-52 (6.5), and HPV-33 (3.5%) [6]. The survey focused on HPV genotyping has discovered that HPV-16 and HPV-58 are the most common types of cervical cancer in 14 provinces of China. Our results show that among the total of 58 CSCC with HPV infection, the two of these cases account for a large proportion. But the most prevalent HPV genotypes in these 54 positive results of ESCC were HPV-16 and HPV-18. In the past 20 years, the reported HPV infection rates of CSCC patients have always been higher than those of ESCC patients form different regions [22, 23]. In our study, we also come to the conclusion of the sample that the HPV infection rate was higher in CSCC than in ESCC (HPV-positive rates: CSCC: 98.3%, ESCC: 59.3%). However, the HPV infection rates in normal epithelium or precancerous lesions did not differ significantly between esophagus and cervix. Our data confirmed that HPV-16 and HPV-18 are the most frequent viral type in both esophagus and cervix. The high-risk HPV types have strongly suggested a possible role of oncogenic HPV infection in carcinogenesis of CSCC and ESCC in Xinjiang [24]. For now and in the time to come, we appeal for more research that could devote to take large-scale investigation in order to explore the distribution of different HPV genotypes in different regions [25] and focus our efforts on improving the popularization rate of early screening and the propaganda of prevention knowledge.

In conclusion, our data show that HPV infection plays an essential role in both cervical and esophageal carcinogeneses. The high-risk types may be positively promoting the occurrence and development in ESCC. High-risk-type HPV-16 was associated with both cervical cancer and esophageal cancer, which play a role in the high-incidence area in Xinjiang. Further studies are needed to elucidate the role of HPV between esophagus carcinogenesis and cervical cancers and study design and analyze the national and regional differences, providing a more accurate assessment of type-specific HPV risk on these cancers.

## Figures and Tables

**Table 1:** HPV prevalence in different cervical and esophageal lesions.

Diagnosis	*N*	High risk					Low risk		
		*n*	(%)	*P*	OR	95% CI	*n*	(%)	*P*
Cervical									
CNOR	40	2	5				7	17.5	
CIN	53	37	69.8	<0.001	43.94	9.44-204.56	7	13.2	0.57
CSCC	59	50	84.7	<0.001	105.56	21.54-517.19	8	13.5	0.59
Esophageal									
ENOR	40	3	7.5				5	12.5	
DYS	26	9	34.6	0.005	6.53	1.57-27.21	4	15.3	0.74
ESCC	54	29	53.7	<0.001	14.31	3.93-52.10	4	7.4	0.41

CNOR: cervical normal; CIN: cervical intraepithelial neoplasia; CSCC: cervical squamous cell carcinoma, ENOR: esophageal normal; DYS: esophageal dysplasia; ESCC: esophageal squamous cell carcinoma.

**Table 2:** Distribution of HPV genotype in HPV-positive cases.

Diagnosis	*N*	High risk						Low risk		
		HPV-16	HPV18	HPV56	HPV59	HPV33	HPV45	HPV6	HPV11	HPV43
		*n* (%)	*n* (%)	*n* (%)	*n* (%)	*n* (%)	*n* (%)	*n* (%)	*n* (%)	*n* (%)
Cervical										
CNOR	9	1 (11.1)	0 (0)	1 (11.1)	0 (0)	0 (0)	0 (0)	1 (11.1)	4 (44.4)	0 (0)
CIN	44	26 (59.1)	5 (11.4)	0 (0)	3 (6.8)	1 (2.2)	1 (2.2)	1 (2.2)	3 (6.8)	1 (2.2)
CSCC	58	37 (63.7)	3 (5.2)	5 (8.6)	1 (1.7)	1 (1.7)	1 (1.7)	1 (1.7)	3 (5.2)	1 (1.7)
Esophageal										
ENOR	8	3 (37.5)	0 (0)	0 (0)	0 (0)	0 (0)	0 (0)	2 (25)	1 (12.5)	1 (12.5)
DYS	13	6 (46.2)	2 (15.4)	0 (0)	0 (0)	0 (0)	0 (0)	3 (23.1)	0 (0)	0 (0)
ESCC	33	21 (63.6)	5 (15.2)	0 (0)	1 (3.0)	0 (0)	1 (3.0)	3 (9.1)	1 (3.0)	0 (0)

**Table 3:** HPV-positive rates of infection types in CSCC and ESCC patients.

	HPV-positive rates (%)	Single-type infection (%)	Multiple-type infection (%)			
			Co-infection	Tri-infection	Quainfection	Seven infections
CSCC (*n* = 59)	98.3 (58/59)	81.0 (47/58)	45.5 (5/11)	18.2 (2/11)	18.2 (2/11)	18.2 (2/11)
ESCC (*n* = 54)	59.3 (33/54)	93.9 (31/33)	100 (2/2)	0	0	0

**Table 4:** Association between infection with HR-HPV and clinical pathological characteristics of ESCC and CSCC patients.

Variables	Cases	HPV infection		*χ* ^2^	*P*
	(*n*).	Positive (%)	Negative (%)		
CSCC patients					
Age (years)					
<50	12	9 (75.0)	3 (25.0)	0.363	0.547
≥50	47	41 (87.2)	6 (12.8)		
Clinical stage					
I	38	33 (86.8)	5 (13.2)	0.050	0.823
II	21	17 (81)	4 (19)		
Differentiation					
High	32	27 (84.4)	5 (15.6)	5.508	0.039
Middle	12	8 (66.7)	4 (33.3)		
Low	15	15 (100)	0 (0)		
Lymph node metastasis					
Positive	19	13 (68.4)	6 (31.6)	4.065	0.044
Negative	40	37 (92.5)	3 (7.5)		
ESCC patients					
Age (years)					
<50	24	12 (50.0)	12 (50.0)	0.238	0.625
≥50	30	17 (56.7)	13 (43.3)		
Gender					
Male	31	19 (61.3)	12 (38.7)	1.685	0.194
Female	23	10 (43.5)	13 (56.5)		
Tumor stage					
I	33	16 (48)	17 (52)	0.930	0.335
II	21	13 (61.9)	8 (38.1)		
Differentiation					
High	17	8 (47.1)	9 (52.9)	1.437	0.487
Middle	22	11 (50.0)	11 (50.0)		
Low	15	10 (66.7)	5 (33.3)		
Lymph node metastasis					
Positive	18	11 (61)	7 (39)	0.596	0.440
Negative	36	18 (50)	18 (50)		

## Data Availability

The data used to support the findings of this study are available from the corresponding author upon request.
